# Causes of Adult Splanchnic Vein Thrombosis in the Mediterranean Area

**DOI:** 10.4084/MJHID.2011.063

**Published:** 2011-12-19

**Authors:** Valerio De Stefano, Tommaso Za, Angela Ciminello, Silvia Betti, Elena Rossi

**Affiliations:** Institute of Hematology, Catholic University, Rome, Italy

## Abstract

The term splanchnic vein thrombosis encompasses Budd-Chiari syndrome (BCS), extrahepatic portal vein obstruction (EHPVO), and mesenteric vein thrombosis.

Risk factors can be local or systemic. A local precipitating factor is rare in BCS, while it is common in patients with portal vein thrombosis. Chronic myeloproliferative neoplasms (MPN) are the leading systemic cause of splanchnic vein thrombosis, and are diagnosed in half BCS patients and one-third of EHPVO patients; the somatic mutation JAK2 V617F is detectable in a large majority of patients with overt MPN, and up to 40% of patients without overt MPN. Inherited thrombophilia is present in at least one-third of patients, and the factor V Leiden or the prothrombin G20210A mutations are the most common mutations found in BCS or EHPVO patients, respectively. Multiple factors are present in approximately one-third of patients with BCS and two- thirds of patients with portal vein thrombosis.

In a few patient series from the Southern Mediterranean area the high prevalence of MPN and thrombophilia as underlying cause of BCS is confirmed, although the data should be considered preliminary. Peculiar risk factors present in the area are Behçet’s disease and hydatidosis; moreover, membraneous webs, typically found in Asian patients, are present in a significant portion of cases.

## Definition

The portal vein is formed by the union of the superior mesenteric and splenic veins. At the porta hepatis, the portal vein divides into the right and left branches, which are segmentally distributed throughout the liver; the terminal portal venules drain into the sinusoids. The hepatic venous outflow travels through the three hepatic veins to the inferior vena cava. The term splanchnic vein thrombosis encompasses occlusions of veins that constitute either the portal vein system or the hepatic veins (Budd-Chiari syndrome, BCS).

BCS is defined as any obstruction of the hepatic venous outflow at any region from the small hepatic veins to the junction of the inferior vena cava and the right atrium. The obstruction can involve small hepatic veins, large hepatic veins, the inferior vena cava, or a combination of these sites. Outflow obstruction can be caused by hepatic veno-occlusive disease (sinusoidal obstruction syndrome) or cardiac disorders associated with right heart failure, which are not included in this definition.^1^

EHPVO is defined as obstruction of the extra-hepatic portal vein. The obstruction may occlude the intra-hepatic portal veins, splenic veins, or superior mesenteric veins. EHPVO also includes the formation of portal cavernomas and the development of portal hypertension, which are associated with long-term disease. Isolated occlusion of the splenic or superior mesenteric vein, as well as portal vein obstruction associated with chronic liver disease or tumor, is not defined as EHPVO.^2^

## Epidemiology

### Budd-Chiari syndrome

The annual incidence of BCS is 0.4 to 0.8 per million individuals in Western countries^3^–^5^ and 0.1 per million in Japan.^3^ BCS has a prevalence of 1.4 per million individuals in Western Countries^5^ and 2.4 per million in Japan.^3^

### Extrahepatic portal vein obstruction

In the 1980s, the estimated annual incidence of portal vein thrombosis was less than 4 per million individuals;^4^ indeed, autopsy studies have shown that portal vein thrombosis is present in approximately 1% of cases – one-third are EHPVO and two-thirds are related to cirrhosis or hepatocarcinoma.^6^

### Mesenteric vein thrombosis

The annual incidence of superior mesenteric vein thrombosis is 2,7 per 100,000 individuals.^7^ Isolated MVT without concomitant EHPVO and splenic vein thrombosis is rare.^8^,^9^

## Clinical Presentation

### Budd-Chiari syndrome

Posthepatic obstruction associated with BCS leads to increased sinusoidal pressure, which may cause perisinusoidal necrosis and eventually liver failure. Hepatomegaly, splenomegaly, right upper abdominal quadrant pain, and ascites occur in the majority of patients.^10^,^11^ Additionally, portal hypertension can develop and EHPVO is concomitant in 14% of cases.^10^

BCS symptoms depend on the extent and rapidity of the hepatic outflow obstruction, as well as liver decompression via a collateral blood flow; accordingly, disease presentation can be classified as fulminant, acute, subacute or chronic.^1^ Fulminant BCS is rare (5% of cases) and is associated with a rapid onset, hepatocellular necrosis, and hepatic encephalopathy. Acute BCS is reported in 20% of patients and is associated with symptoms that last for a short duration, such as ascites and hepatic necrosis, without the formation of venous collaterals. The chronic form of BCS is the most common form as it occurs in 60% of cases, usually with symptoms of portal hypertension and liver cirrhosis.^11^ The remaining 15% of BCS patients are asymptomatic because one patent hepatic vein or large collaterals can preserve the hepatic outflow.^12^ However, in a recent multicentre survey of consecutive cases, the prevalence of asymptomatic BCS was notably lower (3%), and the mortality rate at 6 months was 10%.^13^ After 10 years of follow-up, the overall survival of patients with BCS was 57–62%.^10^,^14^

### Extrahepatic portal vein obstruction

EHPVO presentation can be acute or chronic. Acute thrombosis is characterized by a sudden onset of abdominal pain, no evidence of chronic portal hypertension (gastrointestinal bleeding, ascites, collateral portosystemic circulation, or hypersplenism), and no imaging or ultrasound evidence of portosystemic collateral veins. In the absence of an underlying liver disease, liver function is normal due to a compensatory increase in the hepatic arterial blood flow, as well as a rapid development of collateral veins. If the mesenteric veins are also obstructed, there is a substantial risk of intestinal ischemia and subsequent bowel infarction. EHPVO may also be asymptomatic and can be occasionally diagnosed as the chronic form.^15^ Chronic EHPVO is the late-stage sequela of thrombosis and is usually defined by the presence of a portal cavernoma. The disease is characterised by replacement of the portal vein by fibrous tissue and the development of periportal collateral vessels. Chronic EHPVO can cause portal hypertension with splenomegaly, and the frequency of bleeding of oesophageal varices is high as 12% patient-years.^16^ Other less frequent manifestations of chronic EHPVO include portal cholangiopathy and hepatic encephalopathy.^15^ The overall survival of patients with EHPVO is 54% after 10 years, although in the absence of cancer, cirrhosis, and thrombosis of the mesenteric vein, the survival rate is increased to 81%.^17^ However, the mortality rate of patients with chronic EHPVO and mesenteric vein thrombosis is 5% at one year.^17^

### Mesenteric vein thrombosis

MVT presentation can be acute, subacute, or chronic.^18^ Acute thrombosis is associated with a bowel infarction in one-third of the of patients,^7^ and the mortality rate of MVT is 20%.^7^ In the vast majority of patients, the onset of MVT is characterised by acute abdominal pain. Other common symptoms include diarrhea, nausea, vomiting, and lower gastrointestinal bleeding.^18^ MTV is associated with EHPVO in 65% of patients,^8^ and chronic presentation with no acute abdominal pain and extensive venous collateral circulation is common.^18^

## Risk factors

Risk factors for splanchnic vein thrombosis can be local or systemic, and the latter are influenced by inherited or acquired conditions (Tables 1–2). The combination of multiple concurrent factors is present in 10–46% of patients with BCS^13^,^19^–^22^ and in 10–64% of patients with portal vein thrombosis.^17^,^20^–^23^

BCS is considered primary when obstruction of the hepatic venous outflow tract is the result of an endoluminal venous lesion (thrombosis or web). In contrast, BCS is considered secondary when the obstruction results from the presence of extravascular material (for example a tumor or a parasitic mass) in the lumen or from extrinsic compression (such as abscesses, cysts, tumors). Although the presence of a membraneous web that obstructs the terminal portion of the inferior vena cava is a rare cause of BCS in Western countries, it causes a large majority of cases in Oriental countries.^3^ There is evidence that these occluding membraneous webs are not congenital but are the late-stage sequelae of a previous thrombotic obstruction of the inferior vena cava.^3^

Recent studies from India have shown that isolated inferior vena cava obstruction is nowadays diagnosed in a minority of patients.^24^ Poverty, malnutrition, recurrent bacterial infections, and filariasis have been suggested to be predisposing factors for inferior vena cava occlusion. Thus, improvements in hygienic and sanitary conditions in Eastern patients may partially explain the recent change in BCS pathogenesis.

In Western countries, most BCS patients are females, whereas in Asia the male to female ratio is close to 1.^3^ Use of oral contraceptive is common in Western countries while it is rare in the Eastern countries. A higher prevalence of pregnancy or puerperium associated BCS is found in Asian countries, as such oral contraceptive-related BCS is more common in Western countries than that in Asia.

The leading cause of splanchnic vein thrombosis is myeloproliferative neoplasms (MPN), which are diagnosed in half of BCS patients and one-third of EHPVO patients.^13^,^17^,^20^,^21^,^25^ Until the mid 1990s, the so-called occult MPN was diagnosed on the basis of spontaneous endogenous erythroid colonies (growth of erythroid colonies in the absence of exogenous erythropoietin),^25^ which allowed recognizition of MPN in the early stages. However, this positive result did not fulfil the complete diagnostic criteria for up to 80% of BCS patients and 50% of EHPVO patients.^25^ Recently, the JAK2 V617F mutation has been reported to be the main molecular marker of the Philadelphia-negative MPN. This mutation occur in nearly all patients with polycythemia vera and in about half of the patients with essential thrombocytemia. Thus, this mutation is now recognised as a diagnostic cornerstone.^26^ The close relationship between MPN and splanchnic vein thrombosis has been confirmed by the current one-third prevalence of the JAK2 V617F mutation among patients with BCS and EHPVO (Tables 1-2).^27^,^28^ Patients with splanchnic vein thrombosis that show no additional signs of haematologic disease other than the JAK2 V617F mutation at the time of thrombosis have an overt MPN development rate as high as 52% during the follow-up.^28^

Inherited thrombophilia is found in patients with splanchnic vein thrombosis, although diagnosis of inherited deficiencies of antithrombin, protein C, and protein S is difficult in the presence of liver impairment, which causes a reduced synthesis of the natural anticoagulant proteins.^13^,^23^,^29^,^30^ This difficulty is not present with the factor V Leiden and prothrombin G20210A mutations. Interestingly, a high prevalence of prothrombin G20210A mutation has been consistently reported in various series of patients with EHPVO,^23^,^29^,^30^ whereas factor V Leiden appears to be more common in BCS patients.^13^,^21^ A recent meta-analysis showed a 4-fold increased risk for prothrombin G20210A in EHPVO patients and a 2-fold increased risk for factor V Leiden in BCS patients.^30^

BCS is the most frequent thrombotic complication of paroxysmal nocturnal hemoglobinuria (41% of the occlusive events)^31^ and of Behcet’s disease (26% of the occlusive events).^32^

The known circumstantial risk factors for BCS are pregnancy, puerperium and the use of oral contraceptives.^33^,^34^ A case control study showed that oral contraceptives were associated with a 2.4-fold increased risk of BCS,^34^ although a more current estimation for currently used hormone preparations is needed.

While a local precipitating factor is rare in patients with BCS,^19^,^20^,^21^ it is identified in at least one-third of the patients with portal vein thrombosis.^15^ Inflammatory or malignant abdominal foci, surgical trauma to the portal vein, and liver cirrhosis are among the local causes of portal vein thrombosis (Table 2). EHPVO develops in about 5% of patients undergoing splenectomy, especially in patients with cancer, myeloproliferative disorders or haemolytic anemia.^35^ Reports on the risk factors associated with MVT were mostly anedoctal until recently, when an autopsy series and a population-based study were published.^7^,^8^ The autopsy study stated that abdominal cancer was present in 22% of cases and liver cirrhosis was present in 17%. The population-based study showed a marker of thrombophilia in 67% of patients, a local factor (surgery or inflammation) in 25%, cancer in 24%, and oral contraceptive use in 6% of patients.

### Risk factors in the Southern Mediterranean area

The large majority of reports concerning BCS are from Western countries or Asia (in particular, India and Japan). A few case series of adult patients from countries of the Southern Mediterranean area are available.

In a survey from Israel,^36^ 29 patients with hepatic vein thrombosis were diagnosed between 1955 and 1975. Fifteen of the patients were Jews and 14 were Arabs. In contrast to the Jewish patients, all of whom were adults, the majority of the Arab patients were children below 10 years of age. Primary hepatic vein occlusion was 2.4 times more common among Arab than among Jewish patients. Of the 11 Arab patients with primary hepatic vein occlusion, three had histological changes typical of veno-occlusive disease; whereas in five others, thrombotic occlusion of large hepatic veins or of the vena cava was documented. Although no plant alkaloids could be directly implicated in any of the Arab patients, circumstantial evidence strongly supports such an etiology; all Arab patients originated from small agricultural communities where ancient methods of winnowing, which expose the home-ground wheat to a high risk of contamination by grains containing pyrrolizidine alkaloids, are still in use.

In a series of 22 patients with BCS from Israel,^37^ 10 demonstrated spontaneous erythroid cell growth (45%), in two cases associated with overt polycythemia vera. Seven patients (32%) had protein C deficiency, six patients (27%) had activated protein C resistance, five (23%) had anti-cardiolipin antibodies, five (23%) had antithrombin deficiency, and four patients (18%) had protein S deficiency.

In a series from Saudi Arabia out of 29 patients with BCS,^38^ nine patients had Behcet’s disease (31%); seven had malignancy (24%), eight had antiphospholipid syndrome (27%); of the remaining patients, two had no known cause, one had trauma, one had protein C deficiency, and one had a nephrotic syndrome.

In a large series of 75 patients with BCS from Turkey,^39^ at least one etiological factor was determined in 54 (72%) of them. The etiology could not be defined in 21 (28%) patients. One etiological factor was found in 52% of patients, two in 19%, and three factors in 1%. The most common acquired causes were membraneous webs in 12 patients, (16%), hydatid disease in eight (11%), Behcet’s disease in seven (9%). Overall, thrombophilia (antithrombin, protein C, protein S deficiency, APC-resistance, anticardiolipin antibodies) was detected in 20 patients (26%). Notably, overt MPN were diagnosed only in six patients (8%). In this series the JAK2 V617F mutation was not investigated; therefore, the prevalence of MPNs could be underestimated. BCS was associated with pregnancy in 14% of 35 female patients and with oral contraceptive use in 8%.

In a series of 94 patients with BCS from Egypt^40^ at least one etiological factor was found in 49% of patients, two in 31%, three in 8%, and four in 3%. The JAK2 V617F mutation was found in 29% of 62 patients tested; Behcet’s disease was diagnosed in 12 patients (13%). Overall, antithrombin, protein C, and protein S deficiencies were found in nine patients (9%); out of 64 patients tested, 10 patients were homozygous for factor V Leiden and 24 were heterozygous (52%), whereas prothrombin G20210A was detectable only in 5%. This exceeding prevalence of factor V Leiden could mirror the high prevalence of the mutation in the general population of this geographic area.^41^ A total of 15% of 58 female patients had received hormonal treatment whereas 17% had BCS associated with pregnancy.

A series of 95 patients with portal vein thrombosis (in 25% of cases associated with liver cirrhosis) has been described in Turkey;^42^ nine patients had overt MPN (10%). The prevalence of inherited thrombophilia in this series is not reliable due to the lack of investigation for factor V Leiden and prothrombin G20210A.

## Conclusions

In the Southern Mediterranean area the association of deficiency of natural anticoagulants with BCS is similar to that found in Western countires; unfortunately, the prevalence of factor V Leiden and prothrombin G20210A is described only in one study from Egypt, reporting the presence of factor V leiden in half of the patients. The presence of spontaneous endogenous erythroid growth or JAK2 V617F as reliable signs of MPN has been detected in one-third to half of patients, with a prevalence quite similar to that reported in patient series from Western countries.

Interestingly, Behcet’s disease is the underlying cause of BCS in about 10% of patients from Turkey and Egypt. At variance with series from high-income countries, another cause potentially present in a large portion of patients is hydatidosis. Finally, the presence of membraneous webs has been reported in 16% of patients from Turkey. As noted by the authors,^39^ this prevalence is not as high as in Eastern countries but higher than in Western ones, ideally mirroring the position of this area as a bridge between East and West.

## Figures and Tables

**Table 1:** Causes of Budd-Chiari syndrome (BCS) in adults.

LOCAL RISK FACTORS (%)	% of the patients	SYSTEMIC RISK FACTORS (%)	% of the patients
**- Acquired**		**- Inherited**	
Cancer	6–7	Antithrombin deficiency	2–5
Cirrhosis	8–14	Protein C deficiency	2–9
Abdominal infection	7	Protein S deficiency	3–7
Liver abscess	2	Factor V Leiden	4–26
Inflammatory bowel diseases	3–8	Prothrombin G20210A	3–8
Membranous web	1–4 (West)-30 (East)	**- Acquired**	
**- Circumstantial**		Myeloproliferative neoplasms (MPN)	23–49
Abdominal surgery	2–23	JAK2 V617F (with overt MPN)	57–100
Splenectomy	2	JAK2 V617F (without overt MPN)	44
Abdominal trauma	10	Antiphospholipid antibodies	1–11
		Behcet disease	4–9
		Autoimmune diseases	10–13
		Paroxysmal nocturnal hemoglobinuria	2–19
		Hyperhomocysteinemia	2–9
		- Circumstantial*	
		Oral contraceptives	15–50
		Hormone replacement therapy	14
		Pregnancy or puerperium	4–16

* percentage calculated on the number of women.

The per cent values are the rate ranges from single studies^5^,^9^,^13^,^19^–^22^,^33^ and from revision papers.^25^,^27^,^28^

**Table 2:** Causes of portal vein thrombosis in adults.

LOCAL RISK FACTORS (%)	% of the patients	SYSTEMIC RISK FACTORS (%)	% of the patients
**- Acquired**		**- Inherited**	
Cancer	13–24	Antithrombin deficiency	1–2
Cirrhosis	17–18	Protein C deficiency	1–9
Abdominal infection	10	Protein S deficiency	1–5
Liver abscess	3–5	Factor V Leiden	3–8
Inflammatory bowel diseases	1–4	Prothrombin G20210A	3–22
Pancreatitis	6–19	**- Acquired**	
Cholecystitis	2–7	Myeloproliferative neoplasms (MPN)	6–33
Appendicitis	1	JAK2 V617F (with overt MPN)	78–100
Tuberculous lymphadenitis	3	JAK2 V617F (without overt MPN)	27
Neonatal omphalitis	1–6	Antiphospholipid antibodies	3–13
**- Circumstantial**		Autoimmune diseases	1–4
Abdominal surgery	10–30	Paroxysmal nocturnal hemoglobinuria	1–2
Splenectomy	7	Hyperhomocysteinemia	9–19
Cholecystectomy	3–12	Increased FVIII levels	60
Gastrectomy	3	- Circumstantial*	
Liver transplantation	2	Oral contraceptives	15–30
Abdominal trauma	1– 3	Hormone replacement therapy	3
		Pregnancy or puerperium	2–3

* percentage calculated on the number of women.

The per cent values are the rate ranges from single studies^17^,^20^–^23^,^29^ and from revision papers.^25^,^37^,^28^,^30^
